# Bioengineered Skin Intended for Skin Disease Modeling

**DOI:** 10.3390/ijms20061407

**Published:** 2019-03-20

**Authors:** Maria Sarkiri, Stephan C. Fox, Lidy E. Fratila-Apachitei, Amir A. Zadpoor

**Affiliations:** 1 Department of Biomechanical Engineering, Faculty of Mechanical, Maritime, and Materials Engineering, Delft University of Technology (TU Delft), Mekelweg 2, Delft 2628CD, The Netherlands; marwsarkiri@gmail.com (M.S.); a.a.zadpoor@tudelft.nl (A.A.Z.); 2 Product Development Group Zurich, Institute of Design, Material and Fabrication, Department of Mechanical and Process Engineering, Swiss Federal Institute of Technology (ETH Zurich), Leonhardstrasse 21, 8092 Zurich, Switzerland; sfox@ethz.ch

**Keywords:** bioengineered skin, biofabrication, skin disease modeling, standardization

## Abstract

Clinical use of bioengineered skin in reconstructive surgery has been established for more than 30 years. The limitations and ethical considerations regarding the use of animal models have expanded the application of bioengineered skin in the areas of disease modeling and drug screening. These skin models should represent the anatomical and physiological traits of native skin for the efficient replication of normal and pathological skin conditions. In addition, reliability of such models is essential for the conduction of faithful, rapid, and large-scale studies. Therefore, research efforts are focused on automated fabrication methods to replace the traditional manual approaches. This report presents an overview of the skin models applicable to skin disease modeling along with their fabrication methods, and discusses the potential of the currently available options to conform and satisfy the demands for disease modeling and drug screening.

## 1. General Considerations

### 1.1. Skin

Skin is the largest organ of the human body, covering the whole surface of it. It belongs to the first line of defense, and hence it is an essential part of the immune system. It does not only serve as a barrier preventing the entrance of pathogenic and harmful invaders, but it also hinders the growth of bacteria because of its acidity, while the secretion of antimicrobial peptides—namely defensins—controls the colonization of pathogens when the surface barrier is ruptured [1]. Besides, skin performs many other functions: it protects from the ultraviolet light absorption, it prevents water and electrolytes loss, it regulates the body temperature and it can also serve as a sensation organ via the several nerve endings that can be found in it [1,2].

Skin is composed of two main layers, the dermis and the epidermis. Epidermis is the outer layer of the skin. Its main cell type is the keratinocyte, a specific kind of epithelial cell. It also contains melanocytes, Merkel cells, and Langerhans cells [2]. The thickness of the epidermis differs in the different areas of the body, varying from 0.05 mm to 1.5 mm [3]. The different sublayers of the epidermis, from the innermost to the outermost, are the stratum basale, stratum spinosum, stratum granulosum, stratum lucidum, and stratum corneum. The first sublayer to be produced is the stratum basale, in which the differentiation process of keratinocytes begins, and the other sublayers are then generated. Stratum basale is usually only so thick as one cell, while the thickest epidermal sublayer is stratum spinosum, with a thickness of five to six cells [3]. The outermost layer, namely stratum corneum, is actually a layer of dead cells, and the basic constituent of the skin barrier [4]. Dermis is located under the epidermis. The greatest dermal thickness is approximately 3 mm and can be found on the back [3]. It mostly consists of fibroblasts and the extracellular matrix (ECM) that they secrete. The main component of the ECM is the collagen type I, while also elastin and other types of collagen are found [2,4]. Polysaccharides, proteoglycans and glycosaminoglycans (GAGs) are incorporated in the ECM, as well. Although epidermis is avascular and lacks nerves, dermis contains both blood vessels and nervous tissue, as well as, hair follicles, sebaceous and sweat glands, and lymphatic vessels. Dermis serves as support and as nutrient supply for the epidermis. The two layers are separated via a basement membrane which controls the exchange of molecules between them [4]. Hypodermis lies under the dermis and it contains fibroblasts, macrophages, and adipose cells, as well as nerves and blood and lymphatic vessels. Its main function is fat storage while it also participates in the adaptive immunity [2,5].

### 1.2. Bioengineered Skin 

Bioengineered skin is called an artificially fabricated skin substitute. It is generated by assembling skin cells into a three-dimensional (3D) matrix. The source of cells is a skin biopsy. The isolated fibroblasts are embedded in a 3D matrix, and a period of incubation and cultivation allows their attachment, development, and reorganization in the matrix so that a dermal equivalent arises. Keratinocytes can be then seeded on top of the dermal equivalents to generate a full dermo-epidermal substitute. Cultivation at air-liquid interface results in keratinocytes differentiation and formation of the different epidermal sublayers [6]. Collagen type I usually composes the artificial matrix due to its abundancy in the human dermal ECM, while fibrin is also regarded as a second good option due to its primary synthesis during blood clotting and healing process [7,8]. Synthetic polymers—such as poly(lactic-co-glycolic) acid, polyhydroxybutyrate or poly(ethylene glycol)—are sometimes selected as biomaterials for scaffolds carrying skin cells, due to their good mechanical properties [8,9].

**Figure 1:**
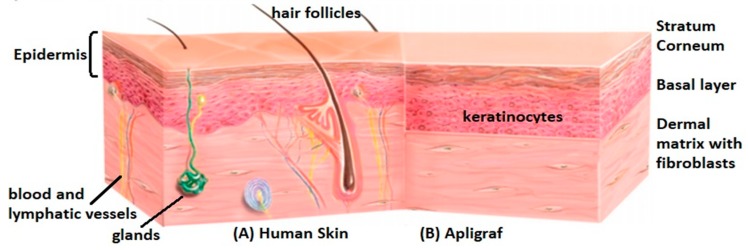
Anatomic differences between native skin (**A**) and an example of dermo-epidermal equivalents, namely Apligraf [11] (**B**). A typical dermo-epidermal equivalent includes a dermal matrix with embedded fibroblasts and the different sublayers of the epidermis, but it lacks hair follicles, sebaceous and sweat glands, blood and lymphatic vessels of the native human skin.

The history of bioengineered skin is already long. Several skin substitutes are nowadays commercialized, while the research continues for improving their quality and properties [5]. However, most of them include currently only the epidermis and/or the dermis, composed of allogenic cells. Furthermore, epidermal equivalents are quite fragile, and keratinocytes proliferate slower in the absence of fibroblasts, while dermal substitutes lack the outer protective skin barrier [7]. Full-thickness dermo-epidermal equivalents are more representative and have been proved to improve and accelerate the healing quality in clinical use in comparison with simple dermal or epidermal analogues [8]. However, they are still a simplified representation of the human skin. Figure 1 depicts anatomical differences between native and a typical paradigm of bioengineered skin. Significant effort is currently focused on the incorporation of other cell types found in skin tissue, to promote vascularization, pigmentation, innervation, and lymphagiogenesis so that the skin model further mimics human skin’s structure and function. A big challenge remains the inclusion of skin appendages, like hair follicles, sweat, and sebaceous glands [10].

### 1.3. Applications of Bioengineered Skin

The initial and basic reason for the development of bioengineered skin has been the acute or chronic wounds mostly caused by burn injuries [12,13]. Autografting has been the gold standard regarding the treatment of these wounds. Autografting includes the transplantation of a healthy autologous skin piece, removed from an undamaged area of the patient [5]. Therefore, it is a slow method of treatment that demands several surgeries, resulting in pain and discomfort for the patient. Additionally, in the case of large wounds, autografts size does not suffice to cover all the injured area [5,13,14]. Allografts is not a fully efficient alternative due to donor shortage and the risk of rejection, while xenografts set risks related to the differences between different species [14]. Trying to overcome these limitations, bioengineered skin substitutes are now used in reconstructive surgery. They are designed to close the wound temporarily or even permanently, restoring the skin barrier and preventing the invasion of pathogens [4,14]. In order to have an efficient and superior alternative to the standard treatment methods, bioengineered skin must be stable, abundant, reproducible, and available in different sizes, while it should undoubtedly serve as a good analogue of native skin [4]. Simple epidermal transplants are sometimes used to restore the skin barrier, while in some cases the graft could also be a simple acellular scaffold, colonized by the patients’ cells after transplantation. Full dermo-epidermal equivalents have been proved to be efficient skin grafts so far, promoting in vivo regeneration [8]. However, the more similar to the native skin, the better the quality and cosmetics of the graft and the faster the initiation of the healing process. Additionally, incorporation of growth factors could control cell behavior and direct the regeneration process after their in vivo time-controlled release [4]. Although reconstruction of skin equivalents from autologous cells would be optimal for the elimination of immune reaction and graft rejection possibilities, it is also tedious, time-consuming, and inconvenient for the patient. The need for prompt transplantation and maximum patients’ convenience has determined the use of allogenic cells, embedded in a pre-fabricated 3D matrix. Although the perfect skin substitute has not been yet developed and progress is continuously pursued, several successful transplantations of bioengineered skin have been achieved, not only in patients with burn injuries but also in people suffering from dermatological disorders, like diabetic ulcers [15].

Although bioengineered skin was initially developed for clinical applications [12,13], it is now also considered as a potential model for preclinical testing of drugs and cosmetic products [4]. Before clinical application of new drugs, their in vivo performance and safety should be predicted. Additionally, cosmetic products must be evaluated, prior to commercialization, not only for efficiency reasons but also for potential allergic reactions or toxic effects. Bioengineered skin is considered a suitable platform for conducting high-throughput screening and assessing pharmaceutical and cosmetic products [16].

**Figure 2:**
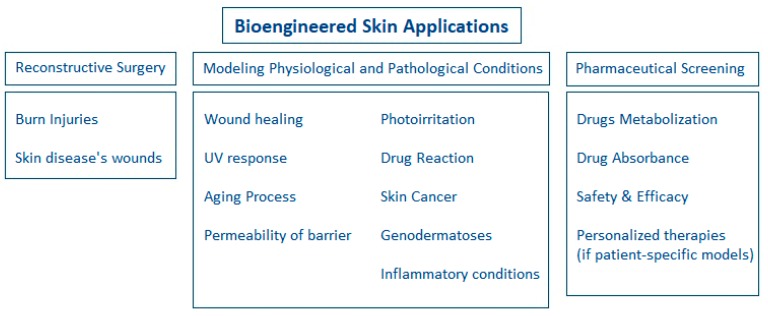
Diagram of the different applications of bioengineered skin: reconstructive surgery, modeling of physiological and pathological skin conditions, pharmaceutical screening.

Furthermore, skin models are now considered beneficial for the modeling of several physiological and pathological skin conditions [4,12,17]. For example, the wound healing process could be reproduced in vitro in the context of regenerative medicine to assess and predict in vivo events. Moreover, the response to ultraviolet irradiation should be assessed to determine its riskiness, considering the interaction between skin exposure and skin cancers. Finally, skin substitutes are now considered useful platforms for the modeling of complicated and severe skin diseases, like psoriasis or skin cancers, as well as for the pre-clinical assessment of potential therapies [4,17,18]. Figure 2 summarizes all the different applications of bioengineered skin nowadays.

Although human skin would be the ideal model to investigate dermatological disorders, studies in human beings are restricted for safety and ethical reasons. Also, the use of animal models is limited not only due to ethical considerations, but also due to the poor resemblance tο native human skin, and therefore the reliability of the research outcome is debatable [19]. Cadavers or biopsies of patients are sometimes used for preclinical studies, but the available specimens might not suffice in size or quantity for an extensive research, while the heterogeneity between specimens coming from different individuals is a downside when reproducibility is required [19]. The in vitro reconstruction of consistent and representative skin models could help overcome these limitations. Utilization of human cells and an extracellular matrix providing the necessary 3D environment for the normal growth, proliferation, and function of the cells enables to achieve a more representative model than 2D cultures or animal models, without posing ethical doubts [19]. Besides, a single biopsy can result in a wealth of cells after their in vitro expansion allowing the fabrication of adequate number of skin models, having the same origin, and thus availability and reproducibility issues are solved. 

Skin substitutes used for skin disease modeling should be consistent to ensure a clear and faithful outcome regarding the effect of the investigated parameters. This demand has to do with the fabrication of the skin models, which has to be fully standardized. Because of the limitations of manual production in terms of standardization, increased focus is placed on the automation of the fabrication methods [2,20]. Besides, rapid research advances would profit from a non-complex fabrication process that could be easily adjusted and modified based on the research needs, while also allowing easy troubleshooting and maintenance. Quick adaptability is important in experimental studies as protocols may be frequently altered, and hence the fast and easy implementation of these changes is both practical and advantageous for rapid progress. 

The objective of this report was to review the skin models applicable to skin disease modeling, along with their available fabrication methods, and to assess their suitability for the specific applications.

## 2. Skin Disease Models and Their Fabrication 

As mentioned above, current applications of bioengineered skin are not restricted to reconstructive surgery but are expanding in the areas of pharmaceutical screening and modeling of physiological and pathological skin conditions. Several types of bioengineered skin can be employed based on the pathogenesis of the disease and the research goals. Besides, the need for reproducible and faithful outcomes, rapid research, and high-throughput screening, has directed the skin tissue engineering community to explore novel fabrication methods of skin models that can satisfy these requirements. 

### 2.1. Skin Disease Models 

In vitro skin models include reconstructed skin substitutes applicable to the investigation of dermatological disorders and their potential medications. They are a promising and emerging alternative nowadays. Nevertheless, their main drawback lies in their structural simplicity, especially in case of complex skin diseases, like psoriasis or skin cancers, the pathogenesis of which results from complicated interactions between cellular or molecular components [21]. Reliable and beneficial modeling of skin diseases demands a robust, standardized, and representative 3D model [22]. A brief overview of available bioengineered skin types applicable to skin disease modeling is presented below while a summary of their advantages and disadvantages is included in Table 1. 

#### 2.1.1. Monolayer Models 

These models consist of a single cellular type: either fibroblasts or keratinocytes. Monolayers of keratinocytes represent the epidermis, while monolayers of fibroblasts represent the dermis. They offer the capability to study the physiology or the pathogenesis of a cell type separately, whereas they partially allow the investigation of cellular interactions by revealing cell behavior in the absence of other cell types. For example, when Chiricozzi et al. studied the effect of a psoriasis-related cytokine, namely IL-17, in keratinocytes monolayers and in full thickness equivalents, they showed a stronger psoriatic effect on the full thickness model [23]. Monolayers lack the 3D environment, which is important to normal growth and function of cells, while they cannot model the interplay between dermis and epidermis [24,25]. 

#### 2.1.2. Reconstructed Human Epidermis (RHS) 

RHS is a skin model consisting of a membrane, made of polycarbonate, in which keratinocytes are seeded and then cultured at air-liquid interface for differentiation and formation of the epidermal sublayers. It is a 3D equivalent modeling the skin barrier but like monolayers, it cannot study the interactions between keratinocytes and other cells found in skin [26]. 

#### 2.1.3. De-Epidermalized Dermis (DED) 

DED models are produced by separating the epidermis from the dermis after biopsy. In addition, an acellular DED can be obtained if the fibroblasts are also removed from the dermis. Keratinocytes are seeded on top of the DED and after cultivation at the air-liquid interface a differentiated epidermis is formed. Seeding of keratinocytes on an acellular DED will result in an epidermal skin model whereas if the DED contains fibroblasts, the seeding of keratinocytes results in a full dermo-epidermal equivalent [27,28] which is more representative to native skin. 

#### 2.1.4. Collagen Hydrogels

These are well-known skin substitutes, having also clinical applications [4]. Human fibroblasts are embedded in a collagen hydrogel, offering the necessary 3D environment, and after a period of incubation and cultivation, they reorganize the dermal matrix and a simple reconstructed dermis occurs. On top of it, keratinocytes are seeded to acquire a full dermo-epidermal equivalent that better applies to the research of skin diseases. High contraction and instability are defects found sometimes in collagen hydrogels [27]. However, these models can be easily and fast produced [24]. Incorporation of more cells found in skin tissue into the reconstructed dermal matrix is required for the modeling of most skin disorders.

#### 2.1.5. Self-Assembled Models 

These models are produced by allowing human fibroblasts to secrete their own extracellular matrix (ECM) in culture, and then keratinocytes of the same source can be seeded on the natural dermis. This method has managed to successfully generate for example psoriatic skin models when diseased skin cells were utilized [24,25]. They are the best representation of either normal or pathogenic skin for in vitro studies due to the absence of synthetic biomaterials and the fully autologous source of cells but their efficacy depends on fibroblasts capability to produce sufficient amount of ECM [24].

#### 2.1.6. Skin-on-Chip Models

**Figure 3:**
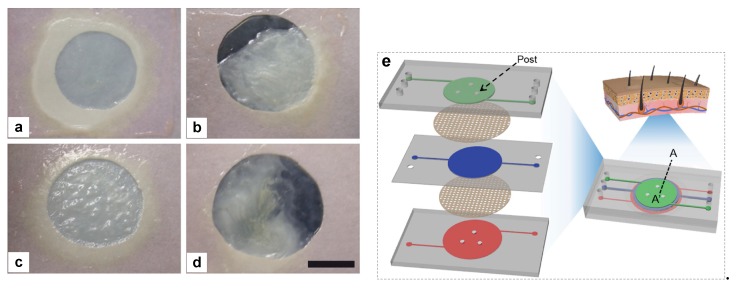
(**a**–**d**) Self-assembled skin model used for psoriatic disease: (**a**) healthy skin substitute; (**b**) psoriatic skin substitute with psoriatic fibroblasts and keratinocytes; (**c**) skin substitute with psoriatic fibroblasts and healthy keratinocytes; (**d**) skin substitute with healthy fibroblasts and psoriatic keratinocytes. Scale bar = 2.2 cm [25]. (**e**) Schematic diagram of a skin-on-chip model consisting of an epidermal layer (top green color), a dermal layer (middle blue color) and an endothelial layer (bottom red color) separated with porous membranes [31]. Right scheme shows the assembled skin-on-chip model, while left scheme illustrates the separate components.

These are more complex structures, useful for the in vitro representation of complex skin diseases. They may differ in structure depending on the incorporated and studied cellular and molecular components but usually the different cell types are cultured in different layers or chambers of the same platform, which communicate and interact with each other through porous membranes, while they also contain microfluidic devices, like micro-valves and mixers, that control the nutrition of the cells as well as the interchange of molecules and fluids between the different layers of the model, trying to mimic the functionality of skin tissue [29,30,31]. Figure 3 illustrates an example of the self-assembled model used for psoriatic disease [25] and a skin-on-chip model combining dermal, epidermal, and endothelial layers [31].

### 2.2. Fabrication Methods 

#### 2.2.1. Manual Fabrication Methods

Production of bioengineered skin has been traditionally conducted manually: from the isolation of cells to the assembly of them in 3D matrix and the in vitro generation of the skin analogues. Various fabrication methods are used for different skin models. 

As already mentioned, in most cases collagen hydrogels are generated, as they resemble the stiffness of skin tissue and resist high amount of water [32]. Plastic compression is a method developed by Brown et al. [33] and optimized later by Brazilius et al. [14] for the prevention of hydrogels high contraction and instability. In this method, the initially thick dermal hydrogels are compressed to reach a final thickness of maximum 1 mm prior to the keratinocytes seeding. The compressive platform can vary in size, thus allowing the fabrication of skin substitutes in several sizes based on the specific need [14,33]. Compression of the hydrogels is carried out manually, and hence it can be laborious. 

In the self-assembled model, fibroblasts are let to secrete their own ECM and a fully autologous dermal equivalent is formed as no biomaterial is added. Use of only autologous cells can result in personalized skin analogues. In most cases, the generated ECM sheets that fibroblasts produce in culture are very thin, and the final dermal matrix results from the manual superposition of more than one sheet together, which is quite a tedious process. The time required for the formation of this skin model is based on fibroblasts capability and quickness to produce their own ECM. Several weeks may be needed to generate sufficient ECM [24,25].

Regardless of the exact method used, the manual character of the processes is a downside. Manual fabrication is not only considered time-consuming and laborious for the researchers, but has also limitations with regard to accuracy and consistency [34]. Pipetting variabilities between different individuals or between different time points have been recorded in relevant experiments. In general, two different individuals cannot be proved to perform precisely the same procedure, while even the same person cannot fully standardize his/her motion. In the case of high-throughput pharmaceutical screening and skin disease modeling, a wealth of similar skin models is required for adequate and reliable experiments to be conducted, and manual fabrication does not ensure the satisfaction of this demand [35].

#### 2.2.2. Automated Fabrication Methods 

The need for time savings, decrease in manual labor and mainly, standardization of the process to ensure reproducibility and consistency of the produced skin models has resulted in efforts to automate the fabrication of bioengineered skin [2,36].

Collagen scaffolds can be fabricated by methods like lyophilization and electrospinning [37]. Fibroblasts can be seeded in these scaffolds for the generation of dermal equivalents. Lyophilization, or freeze-drying, is a dehydration process in which a collagen solution converts into a dry porous collagen scaffold, while in electrospinning, collagen fibers are attracted together forming also porous collagen structures [37], which give cells space to grow, proliferate, and migrate. The formation of the scaffolds is not manual, however the seeding of the cells is still manually conducted and hence tedious and not standardized. 

**Figure 4:**
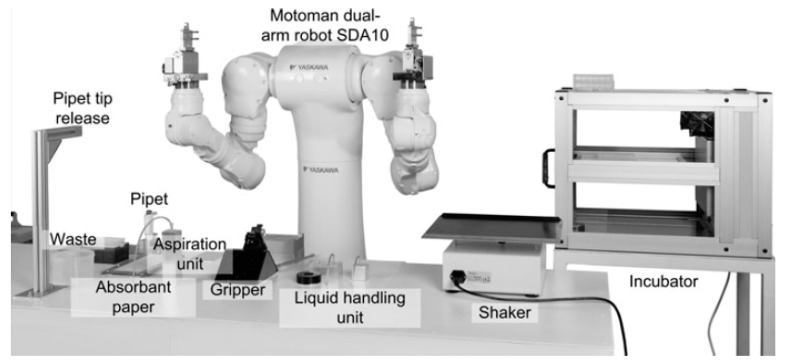
Execution of manual laboratory work by robot [38].

In some laboratories, replacement of humans by robots is currently being attempted [16,38]. Figure 4 illustrates a relevant paradigm, in which a dual-arm robotic system executes pipetting and other biological tasks [38]. The Fraunhofer Group has generated the Skin Factory, in which all the bioengineered skin fabrication steps, ranging from sterilization of the skin biopsies to the assembly of the cells in the 3D matrix, are carried out by a robotic arm with no participation of humans [16,35]. The same group has managed to automatically generate human epidermis models with the use of a robot [16]. This approach has resulted in significant decrease of manual work and the standardization of the production process, as a programmed robot can constantly perform all the steps with fixed speed and fully-controlled motions [16,38]. Despite the automatic character of the process, it remains time-consuming, given that it includes the same steps as in the case of manual fabrication, but human hands are replaced by robotic arms. Another drawback of this method is that robots are barren of human adaptability. The complexity of robotic operation demands constant recruitment of specialized staff for repair, maintenance, or re-programming of the robots for any change in the fabrication protocol, resulting in unavoidable time delays.

3D bioprinting of skin is currently an emerging research area regarding the automated fabrication of skin models [2,20,36]. There are various 3D bioprinting methods, each with its own advantages and limitations. All of them can be categorized according to the main biofabrication approach: the bottom-up and the top-down approach. In the bottom-up approach, small cellular and non-cellular building blocks are arranged in space in a way that a larger 3D biomimetic model is formed. The top-down approach includes seeding of cells along with deposition of suitable biomolecules on larger biodegradable construct so that the cells can eventually form their own 3D matrix that will replace the initial construct. The latter can result in a good biomimetic model, but it demands significant time-investment [2]. 

**Figure 5:**
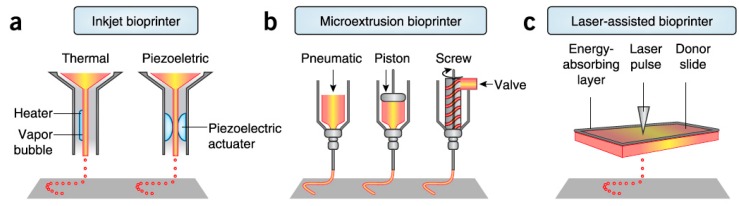
Illustration of three major categories of 3D bioprinting. (**a**) Inkjet bioprinting, in which either heating or electric pulses lead to the injection of bio-ink on the substrate. (**b**) Extrusion bioprinting, in which a mechanical force (pneumatic, piston-driven, or screw-driven) causes the extrusion of biomaterials on the substrate. (**c**) Laser-assisted bioprinting, in which laser pulses on the donor slide result in material deposition on the collector slide [39].

There are three major bioprinting processes which are applied for the fabrication of bioengineered skin: laser-based bioprinting, inkjet bioprinting, and extrusion bioprinting (Figure 5).

- Laser-assisted bioprinting exploits laser energy for the printing. Small droplets of cells are printed on a substrate that can be either a cell culture plate for generation of 2D construct or a scaffold for formation of 3D construct. Precise deposition of the cells in the 3D construct in a high density is achieved by this method while there are no limitations in biomaterials viscosity. Several dermo-epidermal skin substitutes have been already successful fabricated by this method [2,20]. A downside of laser-assisted bioprinting is the relatively low printing speed [2].
- Inkjet bioprinting is based on the ejection of bio-ink droplets on a substrate. Bio-ink contains cell suspension combined with hydrogels or biopolymers. In thermal inkjet bioprinting the droplets are pushed out due to bubbles generated in the nozzle by a heating element, while in piezoelectric inkjet bioprinting, electric pulses result in the droplets ejection [2,34]. Thermal inkjet printing is considered suitable for biological applications as the printed cells are heated at a temperature of less than 10 °C above ambient temperature and for only 2 microseconds, ensuring cell survival during the printing and a later cell viability of about 90%, while piezoelectric approach operates at frequencies that can harm the cells [34]. Inkjet bioprinting can achieve high resolutions and accuracy in deposition but it is efficient only when bio-inks of low viscosity are printed [2]. It has been used for the printing of keratinocytes on top of a previously extrusion-based printed dermal equivalent and it resulted in a very uniform epidermal layer in which keratinocytes quickly and properly proliferated and differentiated throughout the cultivation period [40].
- Extrusion bioprinting is based on the extrusion of a continuous strand of biopolymers or hydrogels, along with cellular components when desired, through a nozzle when mechanical force is applied. Simultaneous printing of cells, biomaterials and growth factors can be achieved in systems of more than one extruder, contributing to the generation of a more complex skin model. This approach is not considered faster than inkjet and laser-assisted bioprinting, but it is suitable for generation of anatomically relevant structures and sizes [2,37]. It also works with high cell density although shear stresses developed in the nozzle may reduce cell viability. Comparison between 3D extrusion bioprinting and manual deposition of skin components revealed the better long-term maintenance of skin equivalents shape and size in case of 3D printing approach [2]. Due to all these advantages, this method could be employed for the generation of dermis which ideally consists not only of fibroblasts and the dermal matrix, but also of several molecular and cellular components. For example, Byoung Soo Kim et al. used this method to fabricate collagen-based scaffolds, including or not polycaprolactone, to form the dermal component of a skin substitute, the epidermal layer of which was later created by inkjet-printing of keratinocytes, as mentioned above [40]. However, this method shorts on resolution capabilities compared to other bioprinting techniques, which affects the precision in cell spatial arrangement [41].

In general, 3D bioprinting offers the capability of massive production of robust and consistent 3D skin models in an automated way, that is faster than the manual or robot-assisted fabrication methods [2,20,36,42]. Besides, regardless of the specific printing technique, 3D printing facilitates the incorporation of several molecules and cell types promoting also pigmentation, innervation, and vascularization, which are important aspects of strongly biomimetic skin equivalents [34]. Scalability is another advantage of 3D-printing approach that benefits high-throughput pharmaceutical screening. Additionally, patient-specific skin substitutes can be still fabricated by printing of autologous cells, contributing to the research of personalized therapies [2,20]. Despite the range of advantages, 3D printers are systems of high-complexity, demanding the recruitment of specialized personnel for sterilization, maintenance, or troubleshooting, while the costs for maintenance and constant supply of materials and tools are not negligible [2].

**Figure 6:**
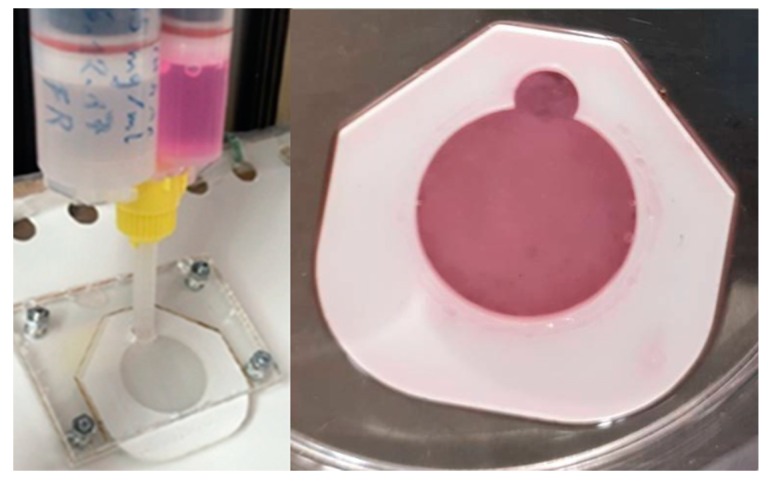
Components of injection molding process [46]. **Left**: Double cartridge, containing high concentration collagen in the left container and fibroblasts suspension in the right container, along with a mixing nozzle in which the contents are mixed and then injected into the encased mold. **Right**: in-mold dermal hydrogel (pink color due to fibroblasts culture medium).

Automated injection molding is another approach, developed and followed by the Product Development Group, Zurich [43,44]. It is based on the plastic compression method of Brazilius et al. [14] but it employs a different technique for avoiding the high contraction of the formed collagen hydrogels. Fibroblasts suspension and high-concentration collagen, instead of low-concentration collagen [14], are mixed and injected into closed molds of 1mm thickness, while cultivation and incubation inside these molds follow to avoid contraction and maintain the size and shape of the dermal hydrogels (Figure 6) [44,45]. A skin-producing machine is used for the automatic blending and injection of the dermal components into the molds, which is considerably faster than manual plastic compression process [43,45]. Consistent dermal equivalents are achieved while scalability can be accomplished with variations in molds size. The next-generation device, in which keratinocytes seeding is also automated, is currently being developed and assessed. Seeding of keratinocytes is carried out without removing the molds, so that in-mold cultivation of dermo-epidermal models follows, aiming to the minimization of manual steps and shape maintenance until final use. The device has been developed such that it offers a friendly user interface and can be easily used by the researchers themselves, while a wide range of settings in terms of flow speed and amounts of injected components allows the immediate adaptation to simple changes in the fabrication protocol without re-programming and processing. However, the time savings of the specific method are restricted to the mixing and injection of skin constituents, while the rest of the process steps are still conducted manually [45].

A comparison of the described fabrication methods is presented in Table 2. The slow production pace and the doubtable consistency of skin models in case of traditional manual fabrication have led to the development of automatic fabrication methods, ranging from robotic systems to 3D printing techniques. Standardization of the fabrication process, scalability, and personalization capabilities are essential to skin disease modeling and high-throughput pharmaceutical screening, while low complexity of the production system might satisfy the needs of rapid research and facilitate use.

## 3. Skin Disease Modeling and Drug Screening

Regardless of their fabrication method and their structure, in vitro reconstructed skin models have been already widely utilized for the representation of several physiological and pathological skin conditions, as well as for the assessment of potential medications and cosmetics products.

### 3.1. Modeling of Physiological Conditions

The modeling of normal skin conditions with the use of bioengineered skin aims to the understanding and prediction of in vivo interactions as well as to the study of molecules that can improve and facilitate them. Optimization of wound healing process, prevention of dangerous impacts of UV irradiation, and delay of aging process are conditions frequently studied with the help of in vitro skin models or skin humanized models, for the understanding of skin tissue function and for the ultimate purpose of a healthier life status. Geer et al. for example fabricated epidermal equivalents, having fibrin as keratinocytes substrate, for the in vitro representation of the wound healing process [6]. Their findings revealed quick initiation of keratinocytes activity and stimulation of in vitro re-epithelization after artificial wounding on the equivalents. They have proposed the use of their skin models for the assessment of different biomaterials in terms of biocompatibility and transport of biomolecules that could be useful to the healing initiation. Besides, Garcia et al. [19] as well as, Guerrero-Aspizua et al. [4] investigated not only the healing process mechanisms but also the response to UV irradiation in skin humanized mice models carrying full dermo-epidermal grafts. Guerrero-Aspizua et al. employed their model to also assess gene-based therapies for defective wound repair and photoprotective compounds against dangerous irradiation [4]. 

### 3.2. Modeling of Pathological Conditions

**Figure 7:**
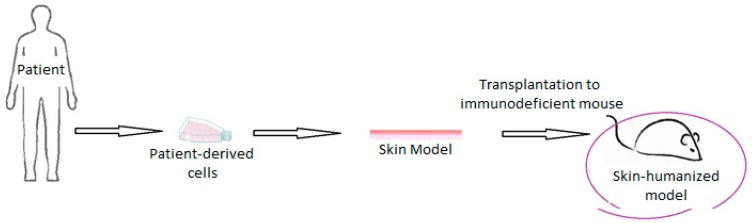
Generation of skin-humanized mouse model: cells are derived from patients or healthy donors, a skin equivalent is in vitro fabricated and cultivated, and then it is transplanted to an immunodeficient mouse.

An emerging application of bioengineered skin is the modeling of skin diseases aiming at the detection and understanding of their triggers and mechanisms of action, thus helping later for the development of an effective therapy [18]. There are two main methods of generating skin disease models, namely by their reconstruction using patient cells, or by addition of genes and molecules to initially healthy bioengineered skin, acting as disease triggers [42]. In both cases, research may be conducted in the in vitro reconstructed skin model or in a skin humanized mouse model, after the grafting of the skin substitute to an immunodeficient mouse (Figure 7). 

Many skin disorders have been modeled with the use of bioengineered skin. For example, Garcia et al. worked together with Meneguzzi’s team in the in vitro replication of epidermolysis bullosa (EB), an inherited skin disease, characterized by fragile skin. Exploitation of patient cells resulted in reconstruction of skin carrying the disease characteristics, while a non-diseased skin equivalent occurred when gene-corrected cells were utilized. Other researchers have also achieved the correction of recessive dystrophic epidermolysis bullosa (RDEB) with the use of gene-corrected keratinocytes in an in vitro study, acquiring also permission for clinical testing [4]. Garcia et al. have also developed bioengineered skin containing cells of patients suffering from Gorlin Syndrome, a skin disorder strongly associated with skin cancer [19]. By grafting it to immunodeficient mice, they made a wealth of skin-humanized mice models for the thorough study of this complex disease. Although in vitro skin models developed under appropriate conditions may enable the study of diseases, skin-humanized mice models or xenotransplantation have been broadly generated and applied for the replication of several genodermatoses, like the Netherton Syndrome, the Kindler syndrome, Mechanobullous disease, and the Xeroderma Pigmentosum, due to scalability capabilities and the better approximation of human skin in comparison with simple animal models [4,21]. Furthermore, these models have been utilized for the investigation of potential gene therapies [4,47]. 

Apart from monogenic skin disorders, Guerrero Aspizua et al. used their skin-humanized mice model to study the pathogenesis and potential therapies for the inflammatory Psoriasis and for a non-epidermal disorder, namely leptin deficiency [4]. In case of psoriasis, they transplanted in mice dorsum a healthy reconstructed skin tissue and injected later lymphocytes and the cytokines IL-17 and IL-22, which have an active role in the specific disease, while they also interrupted the skin barrier via tape-stripping. Gradual evaluation of these parameters allowed them to understand that combination of all these factors results in psoriatic hallmarks, as well as that prior skin injury increases the possibilities for the disease development [22]. 

Bioengineered skin is also considered necessary for the modeling of skin cancer due to its severity and hard treatment. Skin cancer is divided in three types: melanoma, basal-cell carcinoma, and squamous-cell carcinoma [29]. Especially malignant melanoma is a very aggressive category. A suitable skin model for its study requires the incorporation of melanocytes, which abnormally grow and proliferate, passing also through the base membrane. The generation of such a model has been already achieved, showing that the initial location of melanocytes on the skin model and specific proteases released by keratinocytes can influence the degree of melanocytes progression [48]. Besides, as cancer cells receive their nutrients through blood vessels, or even migrate to other body areas through them, vascularization of the skin model is considered as the next goal aiming to the thorough study of the disease, as well as the investigation and in vitro testing of anti-melanoma medications [48].

Additionally, bioengineered skin has been used for the study of herpes simplex virus (HSV) and other viruses. HSV is a well-known and painful skin viral disorder. The virus attacks and passes through the epidermis, and hence 3D skin models are mostly used for the investigation of this first step of infection, as well as to assess recommended anti-viral agents [48]. In vitro skin models of *Candida albicans* have also been generated in order to benefit patients subjected to immunosuppressive therapies, who are easily infected by the specific fungus. The model has managed to detect several involved cytokines, proteases, and transcription factors [48]. 

Reconstructed skin models have been widely utilized in the modeling of several skin diseases, ranging from infections and inflammatory conditions to monogenic skin diseases and skin cancer, having achieved the discovery of plenty of cells and molecules involved. The valuable research of a specific dermatological disorder demands a skin model incorporating all the skin components that participate in the initiation and progression of the specific disease and based on this, different skin models are developed and implemented to the study of different diseases and their potential treatments.

### 3.3. Evaluation of Compounds Safety and Efficacy

In vitro evaluation of newly-developed drugs or cosmetics is essential to prevent negative implications. For example, some chemicals proved to cause photoirritation, a photosensitivity causing irritation to skin in presence of light, whereas some drugs or beauty products have resulted in allergic reactions, and unpleasant or even toxic effects besides their therapeutic activity. Both the efficacy and the safety of new compounds must be verified prior to commercialization and clinical application, firstly for reasons of health but also for the profit of pharmaceutical and cosmetics companies [49]. This evaluation can be conducted in the laboratories of the companies or in external specialized laboratories. In general, the basic tests focus on the potential irritation and corrosion effects of the compounds when in contact with the skin in the presence of light, as well as the absorption degree of the tested substances by the skin tissue. Furthermore, initial assessment is sometimes performed via computer simulations to acquire the first expectations regarding the results [50].

For this purpose, several in vitro skin models have been developed and implemented [51,52], with many of them focusing specifically on photoirritation testing. Especially for dermo-epidermal equivalents, screening of possible irritants or toxic compounds is a typical application [48,49]. Augustin et al. for example, fabricated and used both dermal and full dermo-epidermal substitutes having a collagen matrix to predict the potential in vivo phototoxicity that UV irradiation and specific chemicals can cause [53]. Their research findings were generally comparable with existing in vivo data. In case of dermal models however, the UV penetration was highly increased, a fact explained by the absence of epidermis, the basic skin barrier [53]. Besides, even in the presence of epidermal layer in the reconstructed models, cutaneous penetration of most compounds is found to be higher than in native skin, as Schmook et al. presented in their published work [54]. However, the level of penetration is significantly decreased in comparison with animal skin models or cadaver specimens [54], and the performance is improved not only in terms of permeability, but also of lipid composition, and structural and biochemical characteristics, as supported by Asbill et al. [27] and Monteiro-Riviere, respectively [55]. Roguet et al. used Episkin, a reconstructed epidermis in a collagen substrate, to assess the effect of molecules that are irritating to native skin, and the in vitro reaction was congruent to in vivo findings, thus suggesting that their skin model is a useful tool for the in vitro assessment of several compounds [51]. Besides, Nguyen and Pentoney have discussed specifically about the benefits of bio-printed 3D skin tissue regarding the pharmaceutics preclinical evaluation due to the similarities with native skin in structure and microenvironment, as well as the standardized production [42].

Adverse drug reactions (ADR) refer to any disorder resulting from the use of drugs and can vary in symptoms, underlying mechanisms of action, and severity. ADR do not affect exclusively the skin, but they may have negative impact on other organs as well. Therefore, the bioengineered skin model should also contain other cell types, like immune or liver cells, that are supposed to interact, so that efficient detection of the potential effect of the assessed drugs is achieved [49]. Microfluidics and organ-on-chip technology are proposed to be beneficial for this research due to the capability of recapitulating interactions not only between various cells types but also between the different involved organs [29]. Several skin-on-chip models have been developed and combine the basic skin layers with perfusion pumps or even with other organs, like liver, in order to provide a controlled and similar to skin microenvironment, in which multi-cell and multi-organ interactions are examined, especially in the case of complex diseases [29].

In the field of cosmetics, the employment of skin substitutes, produced by 3D-printing methods, for compounds assessment prior to commercialization is currently pursued by well-known companies. For example, L’Oréal has been collaborating with Organovo for the 3D-printing of skin models which serve as a platform facilitating large scale studies of cosmetic products, and a similar attitude characterizes Procter & Gamble (PNG) [2]. L’Oréal already utilized manually produced bioengineered skin for the evaluation of its products before orienting to the automated fabrication [56], but standardization and scalability capabilities of 3D printing enhance faithful high-throughput screening of the company’s products.

## 4. Discussion

In the previous sections, several studies in which bioengineered skin was implemented for the modeling of physiological and pathological conditions as well as for the investigation and assessment of drugs and cosmetics products have been presented. Skin disorders are numerous, including reactions to cosmetic products or drugs, infections by microorganisms, inherited genetic skin conditions, inflammatory conditions, and skin cancers. In the following paragraphs, the advantages and disadvantages of the existing models and methodologies are discussed based on the research needs.

In most studies in which bioengineered skin is used for skin disease modeling and pharmaceutical screening, the skin models are fabricated manually. Manual production can be tedious and time-consuming for the researchers. The generation of a mature dermo-epidermal construct requires about one month. One to two weeks are usually needed for the dermis maturation and three to four days are demanded for the creation of the dermo-epidermal junction after keratinocytes seeding. For the maturation of the epidermal layer, at least 10 days at the air-liquid interface are required [27,42]. Addition of vascular endothelial cells or lymphocytes to the skin model further increases the preparation time, especially if performed manually, and hence any time savings occurring from the automation of the process would benefit a faster and large-scale research. However, the major downside is the doubtable consistency of the fabricated skin substitutes. Although application to reconstructive surgery does not demand a wealth of identical skin substitutes, in case of high-throughput drug screening and reliable skin disease modeling, consistency is a major requirement [2,16].

Robotic systems and 3D printing are the main current approaches towards the automation of skin models production. Although standardization of the fabrication process is achieved by robotic systems, their complexity and slow production do not facilitate large scale studies and rapid research [16,38]. On the other hand, 3D bioprinting is an emerging fabrication method allowing automated, standardized, massive, and faster production of skin equivalents [2,20]. Despite all the capabilities, 3D printers suffer from high-complexity and high maintenance costs. Recent studies investigate the performance of automated injection molding in terms of standardization capabilities, personalization opportunities, cost-effectiveness, and ease of use. This method has been automated and already examined in the fabrication of stable and consistent dermal equivalents. The simplicity of the user interface and of the operation principle allows the injection by non-specialized staff [43,45]. The main disadvantage of the process is that the prior preparation of cells and biomaterials, as well as the in vitro cultivation remain manual and tedious [43,44]. Nevertheless, it is a promising method for the fabrication of consistent skin substitutes in an automatic, cost-efficient, and non-complex way.

Several skin models, ranging from 2D monolayers to complex 3D skin equivalents have been developed [23,24] or even commercialized [57]. More weaknesses are encountered with the monolayers as they fail to study the interactions between keratinocytes and fibroblasts [23,58,59,60]. The use of collagen hydrogels in skin tissue engineering has been established for many years due to the relatively easy production and continuous availability, as a single biopsy can result in a wealth of skin models after in vitro cultivation of cells [24]. Besides, the plastic compression method [14,33] has managed to eliminate the high contraction and instability of collagen hydrogels, making them even more attractive models [14,24]. On the other hand, the self-assembly approach can offer perfectly autologous skin substitutes, by allowing fibroblasts to secrete and organize their own ECM and hence it facilitates the development of personalized therapeutic strategies. However, the time-consuming character of the procedure does not meet the needs for rapid research and large-scale studies [24]. 

In any case, all skin tissue components that are necessary for the recapitulation of a skin disease should be included in the models. Melanoma, for example, demands also the incorporation of melanocytes and vascular endothelial cells [48], while immune cells play important role in the initiation of psoriasis [61]. 3D bioprinting offers the capability to incorporate various cells and molecules, while researchers have also manually produced models including vascular endothelial cells, immune cells or melanocytes [24,48,62]. Similarly, assessment of pharmaceutics does not benefit from a simplified model of native skin. Full dermo-epidermal equivalents are more widely used in comparison with single-layered models but for complicated cases, like adverse drug reactions for example [49], in which several organs may be the acceptors of the side effects, even these models do not suffice. Here, organ-on-chip technology represents a useful tool, allowing multi-cell and multi-organ modeling [29,30]. Microfluidics and organ-on-chip technology in combination with tissue engineering provide plenty of opportunities and capabilities in disease modeling in general [29].

## 5. Conclusions

Skin diseases demand thorough and continuous investigation, firstly for their understanding and at a later stage for their efficient treatment. Bioengineered skin represents a promising model for the thorough research of pathological skin conditions, as well as of drugs efficacy and safety, overcoming limitations of animal or other models. Especially the research of highly complex diseases, like psoriasis or skin cancer, demands standardized as well as structurally and functionally suitable skin models in order to ensure reliable outcomes, while the high-throughput pharmaceutical screening of potential medications requires lasting availability and scalability. To this aim, the fabrication method of skin models should be automated, standardized, cost-effective, and as non-complex as possible to facilitate easy use and rapid research, while it should allow the incorporation of several cellular and molecular components and the generation of personalized skin models. Significant progress has been achieved by different fabrication methods such as 3D bioprinting and the automated injection molding while the emerging organ-on-chip and microfluidic technologies can further help in mimicking the more complex skin disease systems. Thus, the field provides strong potential for further adaptation and improvements of the current skin models depending on the specific application and research questions addressed. A significant impact of these advances on the understanding and treatment of skin diseases is foreseen.

## Figures and Tables

**Table 1:** Different skin models applicable to skin disease modeling, along with their advantages and disadvantages.

Skin Model	Cells	Matrix	Advantages	Disadvantages
Monolayer models	Keratinocytes or fibroblasts	-	Differentiated epidermis	2D environment, no cellular interactions
Reconstructed human epidermis	Keratinocytes	Polycarbonate	Differentiated epidermis, 3D environment	No cellular interactions
De-epidermalized dermis	Fibroblasts or fully acellular	Natural ECM	3D environment, dermo-epidermal equivalent after keratinocytes seeding	Keratinocytes absence, limited availability
Collagen hydrogels	Fibroblasts (embedded in collagen hydrogels), keratinocytes (seeded on top of hydrogel)	Collagen I (can be combined with GAGs, chitosan or other collagen types)	3D environment, dermo-epidermal equivalent, availability, easy production	No native ECM, contraction of hydrogels
Self-assembled models	Fibroblasts (embedded in collagen hydrogels), keratinocytes (seeded on top of hydrogel)	Natural ECM	3D environment, dermo-epidermal equivalent, fully autologous skin model	Slow and tedious process
Skin-on-chip models	Fibroblasts, keratinocytes, endothelial cells, other organs’ cell types	Porous membranes, scaffolds, or other	3D environment, interactions between different cell types or organs	Complex systems, no native ECM

**Table 2:** Available fabrication methods of skin models along with their potentials and limitations.

Fabrication Method	Potentials	Limitations
Manual fabrication	Incorporation of several cells/molecules, personalization opportunities, fast adaptation to research needs	Slow and tedious process, non-standardized method
Fabrication by robots	Incorporation of several cells/molecules, personalization opportunities, standardized production	Slow process, high-complexity and decreased adaptability
3D bioprinting	Incorporation of several cells/molecules, personalization opportunities, standardized production, faster process	High-complexity and decreased adaptability, expensive
Automated injection molding	Personalization opportunities, standardized production, non-complex process, faster than manual or robotic production	Still slow process, validated only for dermis fabrication yet
